# Paying for Happiness: Experimental Results from a Large Cash Transfer Program in Malawi

**DOI:** 10.1002/pam.22044

**Published:** 2018-02-09

**Authors:** Kelly Kilburn, Sudhanshu Handa, Gustavo Angeles, Maxton Tsoka, Peter Mvula

## Abstract

This study analyzes the short‐term impact of an exogenous, positive income shock on caregivers’ subjective well‐being (SWB) in Malawi using panel data from 3,365 households targeted to receive Malawi's Social Cash Transfer Program that provides unconditional cash to ultra‐poor, labor‐constrained households. The study consists of a cluster‐randomized, longitudinal design. After the baseline survey, half of these village clusters were randomly selected to receive the transfer and a follow‐up was conducted 17 months later. We find that the short‐term impact of household income increases from the cash transfer leads to substantial SWB gains among caregivers. After a year's worth of transfers, caregivers in beneficiary households have higher life satisfaction and are more likely to believe in a better future. We examine whether program impacts on consumption, food security, resilience, and hopefulness could explain the increase in SWB but do not find that any of these mechanisms individually mediate our results.

## INTRODUCTION

The importance of income for individual subjective well‐being (SWB), often described as “happiness” or “life satisfaction,” has been debated for decades (see, e.g., Easterlin, 1974; Easterlin et al., 2010; Frijters, Haisken‐DeNew, & Shields, 2004; Stevenson & Wolfers, 2008). In richer, more developed countries, income is positively correlated with happiness but with diminishing returns (Frey & Stutzer, 2002). At lower levels of income and in low‐income countries, however, there is a stronger linear relationship suggesting that income is more important for happiness when basic needs are yet to be fully satisfied (Deaton, 2008; Graham & Behrman, 2010). Poverty‐targeted social protection programs that have a direct impact on income may therefore also increase SWB. Nevertheless, existing government‐run social protection programs in low‐income countries rarely collect and analyze SWB data so limited evidence exists to support this relationship. This paper uses data from an experimental study of Malawi's Social Cash Transfer Program (SCTP) to examine how income affects happiness amongst household caregivers. It uses measures of SWB that capture concepts of life satisfaction, relative wealth, and future expectations.

But should public policies be judged on how they influence the SWB of beneficiaries? A report by The Commission on the Measurement of Economic Performance and Social Progress (Stiglitz, Sen, & Fitoussi, 2009) recommends SWB as part of a core set of indicators to measure societal economic and social progress, a recommendation that was subsequently endorsed by the European Commission (EU Commission, 2009), and which has led to SWB measures being integrated into national statistical systems such as the annual European Surveys of Income and Living Conditions. Equating quality of life (QoL) or well‐being to the simple command over marketed resources as reflected in income or consumption is limiting for several reasons. First, some essential resources that determine QoL are not marketed. Second, the determinants of individual well‐being often relate to one's life circumstances, including such elements as exposure to risk, personal safety, environmental degradation, and social connectedness, which are not reflected in income‐based measures. Finally, resources are more accurately seen as means that are transformed into well‐being, and individuals have different capabilities to make these transformations. To the extent that the objective of public policy is to deliver social and economic progress in a sustainable manner, the impact of policy interventions on these dimensions is clearly relevant and appropriate. Moreover, the idea that the individual is the best judge of his or her own condition is a long‐standing one in philosophy and other disciplines (Stiglitz, Sen, & Fitoussi, 2009). Incorporating subjective welfare indicators into social policy evaluations can thus complement existing objective measures and provide a deeper understanding of how policies affect QoL across dimensions other than economic or material.

This paper contributes to the literature by providing one of the first rigorous assessments of the impact of a cash transfer program on SWB. A recent review suggests that over 800 million people in the developing world are now reached by some form of state‐sponsored cash transfer (Honorati, Gentilini, & Yemtsov, 2015). In Sub‐Saharan Africa, in particular, there has been a rapid increase in such programs with a doubling (from 20 to 40) in the number of development‐oriented cash transfer programs over the last six years. Evidence from these African programs shows promising effects on schooling (The Kenya CT‐OVC Evaluation Team, 2012a), consumption (The Kenya CT‐OVC Evaluation Team, 2012b), food security (Hjelm, 2016), and even economic productivity (Handa et al., 2016). More recent attention, however, has focused on the effect of cash transfers on subjective assessments and experiences to understand the broader, more holistic impact of these programs on well‐being. Qualitative evidence from programs across Sub‐Saharan Africa and the Middle East reveals a predominantly positive psychosocial impact (Samuels & Stavropoulou, 2016) and that improved psychosocial well‐being may further contribute to positive impacts in other domains such as decisionmaking (Attah et al., 2016). However, quantitative evidence on the relationship between social cash transfers and happiness is limited and inconsistent. A study from Mexico found a dissonance on objective and subjective welfare; increased income for households in the Mexican Oportunidades program did not translate into a greater sense of well‐being (Rojas, 2008). In Kenya, however, a program operated by the NGO GiveDirectly, which provides a large one‐time payment, did result in an increase in subjective measures of well‐being in addition to objective consumption measures (Haushofer & Shaprio, 2016).

This experimental study consists of a cluster‐randomized design where households in village clusters (VCs) were randomly assigned to either the treatment or control group after the baseline survey. We use two rounds of longitudinal data—collected at baseline (pre‐program in 2013) and after 17 months (at the end of 2014). The random assignment to treatment and control status provides exogenous variation in income that allows us to identify the impact of such increases on SWB.

We find that increases in household income from the cash transfer have substantial SWB effects among caregivers in the short term. There are large, significant effects of treatment on both life satisfaction and future outlooks, which are robust across empirical specifications. Specifically, after about a year's worth of transfers, caregivers in beneficiary households score higher on the QoL Scale (0.5 SD) and are more likely to believe in a better future in two years (around 20 percentage points) using panel data specifications. Moreover, we examine whether other program impacts on consumption, food security, resilience, and hopefulness explain the total effect of the program but do not find that any of these mechanisms individually mediate our results.

## BACKGROUND

SWB can be defined as an individual's evaluation of his or her life from both emotional and cognitive perspectives (Diener, Lucas, & Oishi, 2009). Emotional well‐being is an experiential concept, highlighting the range of emotions that affect an individual's present sense of happiness (Kahneman & Deaton, 2010). The cognitive perspective is usually defined as life satisfaction, which refers to a person's global assessment of their life in regard to whether they find life pleasant or fulfilling (Diener et al., 1985). Kahneman and Deaton (2010) find that both emotional well‐being and life satisfaction rise with income, but that the relationship is stronger for life satisfaction while emotional well‐being is more closely related to individuals’ health and relationships. In practice, surveys such as the Gallup World Poll tend to capture more of the cognitive, life satisfaction aspect of SWB. The prevalence and generality of life satisfaction measures means they are appealing to researchers and policymakers alike (Dolan, Layard, & Metcalfe, 2011).

Traditionally, economists have been critical of these life satisfaction measures because self‐reports are assumed to be unreliable signals for individuals’ underlying preferences and constraints that affect actual behavior. Instead, they have relied on revealed preference analysis, examining observable consumption and investment behavior with the underlying assumption that these measurable choices better reflect the set of unobservable trade‐offs of preferences and constraints (Graham & Behrman, 2010). However, people habitually make inconsistent choices that are out of alignment with their own happiness, departing from the standard model of the rational economic agent (Kahneman, 2003). Poverty can additionally increase irrational decisionmaking by increasing the level of competing demands that an individual must process, thereby testing the limits of one's cognitive capacity (Gennetian & Shafir, 2015). Evidence from developing country contexts, for example, finds that people living in poverty consistently fail to participate in market opportunities and make myopic decisions, such as borrowing at very high interest rates (Banerjee & Duflo, 2012; Banerjee & Mullainthan, 2010). Revealed preference analysis is therefore limited in providing an explanation of other factors influencing important choices, such as poor self‐control and constraints like poverty that might result in perverse choices (Gennetian & Shafir, 2015; Graham & Behrman, 2010).

In theory, collecting subjective data allow researchers to test fundamental economic assumptions because subjective data directly capture well‐being (Frey & Stutzer, 2002). Although evidence has confirmed that income is an important determinant of SWB, using income exclusively to evaluate welfare may overvalue policy impacts. There are other human needs and values that cannot be directly bought or enriched with income, such as emotional support and personal relationships as well as autonomy and human development. Moreover, focusing solely on income neglects the fact that income may not be used efficiently and that well‐being could depend more on relative rather than absolute consumption (Rojas, 2007). Alleviation of income poverty might not be enough to increase an individual's overall sense of well‐being if other dimensions of their life are going poorly. As Rojas (2015) describes in his “subjective well‐being approach,” the goals of poverty alleviation programs may be compromised if dissonances emerge between subjective and objective measures. Policies that only improve people's lives in terms of absolute income may not lead to successful transitions out of the poverty cycle since well‐being involves other aspects such as work, relationships, and communities. Thus, well‐being is multi‐dimensional and the measurement and assessment of alternative measures within social programs evaluations can complement objective measures to provide a better picture on the effect of policies across more dimensions than the economic one.

The use of SWB metrics, however, is by no means the only alternative well‐being measure used in policy evaluation. Other alternatives to income include objective composite well‐being indices such as the Human Development Index, the capability approach, expected utility functions, and individual preference measures such as the “equivalent income measure” (Decancq & Neumann, 2014). Evaluations of such approaches, however, have found that these different measures do not have strong overlap and identify disparate groups of individuals as worse off (Decancq & Neumann, 2014; Laderchi, Saith, & Stewart, 2003). While this suggests that no one metric is superior in capturing all aspects of individual well‐being, subjective measures are an attractive choice to complement objective measures given their frequent use, interpretability, and ease of incorporating them into surveys (Dolan, Layard, & Metcalfe, 2011).

Unlike objective metrics, however, SWB relies on individual interpretations of questions, which could make interpersonal comparisons difficult if individuals’ perceptions are widely different (Beegle, Himelein, & Ravallion, 2012). Nonetheless, bias can be reduced with econometric techniques that control for unobserved heterogeneity among individual responses that affect reference points (Graham & Behrman, 2010). Moreover, recent findings indicate that differences in reference points in subjective survey data present little bias in relative well‐being data (Beegle, Himelein, & Ravallion, 2012), and measures of life satisfaction have been validated as a significant correlate across other measures of well‐being including economic, psychological, and physiological ones (Dolan, Peasgood, & White, 2008; Kahneman & Kruger, 2006). Therefore, the use of SWB metrics has become increasingly valued among economists and policy scholars for the purpose of measuring individual and social welfare (e.g., Dolan, Layard, & Metcalfe, 2011; Kahneman & Kruger, 2006; Rojas, 2015).

Despite the growing use and acceptance of subjective data, literature on well‐being and income rarely establishes causality because survey data are usually missing the exact timing of changes in income and happiness, raising concerns about reverse causality. A few studies, however, have been able to test this causally by utilizing natural exogenous variations in income. For instance, Frijters, Haisken‐DeNew, and Shields (2004) use the reunification of Germany to show that income increases for East Germans resulted in lasting gains on individual life satisfaction, while Gardner and Oswald (2007) use data on lottery winners in Britain to show that mid‐size winnings result in better psychosocial health for winners compared to either those without wins or with smaller wins. The lack of experimental data, however, is a consequence of the difficulty in running large‐scale experiments where researchers randomly allot cash to different groups (Gardner & Oswald, 2007).

This paper fills this important gap in the literature by exploiting the randomized study design of an unconditional cash transfer program in Malawi to measure the causal impact of income increases on SWB in short term. Experimental evaluations of unconditional cash transfer programs have recently started to measure SWB. In Kenya, GiveDirectly, an NGO that primarily provided large, one‐time unconditional cash payments to poor households, found that the program had strong positive effects on happiness and life satisfaction measures for recipients after nine months, about the same amount of time as this study (Haushofer & Shapiro, 2016). However, as reported in a working paper, they find larger negative spillover effects for neighbors in the same villages who did not receive the transfer, and that effects for beneficiaries diminished after the study ended, suggesting hedonic adaptation to their new circumstances (Haushofer, Reisinger, & Shapiro, 2015). In contrast to the small, every other month payments from the Malawi SCTP that are intended to continue over a long duration, the GiveDirectly program distributed large transfers (corresponding to around two years of per‐capita expenditure) in a relatively short time frame (either in a lump sum or over nine monthly installments). Although such differences in the programs might affect long‐term results, we do not have data for this study to test for hedonic adaption.

Instead, this study focuses on the short‐term impacts of the Malawi SCTP to provide new evidence on the extent that these large‐scale, government‐run unconditional programs affect SWB. The literature on similar government‐run cash transfer programs and happiness is small. Beyond the previously mentioned Opportunidades study (Rojas, 2008), non‐experimental evidence from Brazil and South Africa suggests that governmental cash assistance programs had a positive effect on life satisfaction for older adults; however, the study could not directly attribute well‐being increases to income (Lloyd‐Sherlock et al., 2012). Some cash transfer experiments have examined the relationship between income and other non‐objective well‐being outcomes such as psychosocial health. For example, a cash transfer experiment in Malawi finds that both unconditional and conditional arms improve the mental health of adolescent girls (Baird, de Hoop, & Ozler, 2013), although a few studies from cash assistance programs in Uganda have not found significant results (Blattman et al., 2016; Green et al., 2016). Besides cash transfers, other poverty alleviation programs in developing countries have mostly found positive evidence on psychosocial measures including a multi‐faceted graduation program (for which cash assistance is a component) (Banerjee et al., 2015).

Missing from this literature, though, is evidence on the channels through which cash transfer programs affect SWB. As most programs lead to improvements in objective outcomes such as per capita food expenditures, increased food consumption, and reduction of food insecurity is often recognized as the most likely pathway. The theory of change proposed in a recent review of cash transfer programs from the Overseas Development Institute also suggests cash transfers may increase recipients’ feelings of self‐esteem, pride, inclusion, and hopefulness through mechanisms such as increased non‐food consumption, reduced dependency on others, and the ability to save and plan for the future (Bastagli et al., 2016). Therefore, in addition to the direct impacts of the Malawi program on SWB, we examine whether program impacts on other measures help explain SWB results including consumption, food security, ability to manage shocks, and perceived financial security.

## THE MALAWI SCTP

The Government of Malawi's (GoM's) SCTP is an unconditional cash transfer program targeted to ultra‐poor, labor‐constrained households. The main objectives of the program are to alleviate hunger and poverty among households and to improve children's well‐being and human capital through education, nutrition, health, and household productivity. The program began as a pilot in the Mchinji district in 2006, and since that time, the program has expanded to 18 districts and reaches 756,000 individuals in 175,000 households as of May 2016.

SCTP beneficiary selection is made through a community‐based approach with oversight provided by local and national government. Appointed community members are responsible for identifying households that meet the eligibility criteria of being ultra‐poor and labor constrained. After further screening of households identified by the GoM, including a proxy means test to meet the ultra‐poor eligibility condition, the recipient list is generated. The program's goal is that these lists target the bottom 10 percent of each community (Malawi SCTP Evaluation Team, 2015). An early evaluation of the Malawi SCTP in Mchinji confirms that recipient households live in extreme poverty and have higher dependency ratios than other poor households (Miller, Tsoka, & Reichert, 2008). Additionally, household heads tend to be older (above 60) and upwards of 80 percent of households are missing at least one prime‐age adult, highlighting their particular vulnerability to the impacts of HIV/AIDS (Handa et al., 2013).

The SCTP targeting criteria is very similar to other large national programs in sub‐Saharan Africa. Table A1 in the online Appendix^1^ shows the average age, proportion female, and average consumption of beneficiaries from five national programs in the region. Program beneficiaries are overwhelmingly elderly (average age of 60) females (ranging from 60 percent in Zambia to 84 percent in Malawi) and extremely poor households (living at or below one dollar per person per day).

The SCTP provides a monthly unconditional cash transfer to eligible households, which varies depending upon the number of members in the household. Table 1 shows transfer amounts in Malawi Kwacha that were in use at the time of baseline and follow‐up data collection. Transfer values were subsequently increased in May 2015. Using administrative data on transfer payments received by households in our study, we calculate that the average value of the transfer is 18 percent of pre‐program consumption. Payments are made every other month in cash at a local pay‐point (typically at a central location in the VC) and at the follow‐up survey we administered a short module to understand program operations and beneficiary perceptions and expectations around the program. Results from this survey show that 88 percent of households received a payment in the last month and all households had received a payment within the last two months. In addition, 90 percent of people walked to the pay‐point and 70 percent spent two hours or less in travel time to and from the pay‐point. Only 3 percent reported missing a payment sometime in the past.

**Table 1:** Structure and level of transfers in Malawi Kwacha

	Transfer levels prior to May 2015
1 Member	1,000
2 Members	1,500
3 Members	1,950
4+ Members	2,400
Each member below age 21^a^	300
Each member below age 30^a^	600

*Source*: Malawi Social Cash Transfer Program Midline Impact Evaluation Report (2015).

^a^These top‐ups are provided to support school enrollment, but payments are not conditional on enrollment.

The initial enrollment into the program is done for a three‐year period at which time households are to be recertified. In practice, though, this recertification has only been done once in two other districts since the program expanded in 2008 and there is currently no concrete recertification plan as program staff is occupied with the scale‐up of the program. When asked how long they expect to receive payments from the program, 40 percent of recipients reported two to five years, 18 percent reported for the rest of their life, and 35 percent stated they did not know. In informal conversations held during field visits by the study team, it also appeared that recipients viewed the transfer as long‐term income support rather than a short‐term, transitory windfall, and we thus expect impacts to reflect this perception.

## STUDY DESIGN

Data come from an impact evaluation of Malawi's SCTP led by UNC‐Chapel Hill's Carolina Population Center and University of Malawi's Center for Social Research. The evaluation was designed as a cluster‐randomized, longitudinal study with a baseline and two follow‐up surveys. Our present study uses the baseline survey conducted in mid‐2013 and the first follow‐up survey conducted in late 2014 through early 2015. The household survey is the main survey instrument, covering a comprehensive list of topics including household composition, consumption, economic activity, education, health, time use, and subjective welfare, among others. A qualitative component also includes in‐depth individual interviews with the caregiver and one youth from 16 treatment households selected using a stratified sampling approach. The study has IRB approval from both the University of North Carolina (IRB Study No. 14–1933) and Malawi's National Commission for Science and Technology (IRB Study No. RTT/2/20).

### Randomization

The impact evaluation was designed around the GoM's plans to extend and expand coverage of the SCTP within Malawi, starting in 2013. In order to integrate the impact evaluation with early expansion plans in 2013, two districts, Salima and Mangochi, were chosen for the evaluation study. Random selection was included at all possible levels, including the two smaller levels within these districts, Traditional Authorities (TAs) and VCs. First, two TAs in each district were randomly selected to participate in the evaluation study, and the GoM subsequently carried out program targeting based on established operation guidelines. The targeting procedure generated eligibility lists in each VC in the designated study sites. Within each TA, VCs were ordered randomly by the study team using the random number generator in excel, and these randomly ordered lists were provided to the government. After the baseline survey was completed, the lists were taken to the District Commissioner's Office in each district where a public coin toss was conducted to determine whether the top half or the bottom half of the list of VCs in each TA would enter the program first, with the remaining VCs entering at a later date. In each district, the District Commissioner designated which half of the list was heads and which was tails, and the result of the coin toss indicated the treatment group. Ultimately, 14 clusters were assigned to the treatment arm and the remaining 15 to the control arm.

### Sampling

**Figure A1:**
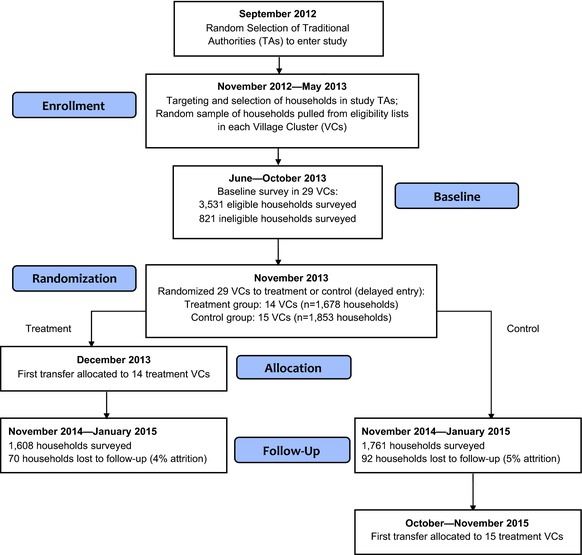
Household‐Level Study Flow Chart for the Malawi SCTP.

The study team computed power for the three key program outcomes of consumption, school enrollment, and child nutritional status using intra‐class correlation estimates from the most recent Malawi Demographic and Health Survey for nutrition and the latest Malawi Integrated Household Survey for consumption and schooling. These calculations led to a sample size of 3,500 households in 29 VCs for an average of 121 households per cluster. Within each VC, households were randomly sorted using a random number generator, and the first 122 households were selected for inclusion in the study. The final sample for the study was 3,531 households (1,852 in control VCs and 1,678 in treatment VCs), approximately 47 percent of all eligible households from the four TAs (see Appendix Figure A1, 2 for a flow diagram of participants). The design was ethically feasible because the program did not have the financial resources to reach all households in program districts immediately. In addition to the eligible households, 821 ineligible households were randomly selected from the same study locations (using a random walk) in order to conduct a targeting and local economy assessment. These households were only surveyed at baseline. We show these data in our baseline analysis to offer some context for our study sample.

The quantitative baseline survey was administered over several months from June to September 2013 to the study sample. The follow‐up occurred at the end of 2014 and concluded in February 2015. Beneficiary households had received five or six cash payments at the time of follow‐up data collection. Each payment accounted for two months so results can be interpreted as one‐year impacts of the program. An important note about the follow‐up is that this survey was conducted in Malawi's lean season while baseline was conducted after the harvest. There was a significant decline in consumption of around 25 percent for both study arms at follow‐up, on par with regional consumption fluctuations between the same time periods in Malawi's 2010 Integrated Household Survey. However, the SCTP appears to be protective for beneficiary households during these seasonal changes as evidenced by greater average consumption across a number of food and nonfood categories (Malawi SCTP Evaluation Team, 2015).

## DATA

### Baseline Balance and Attrition

To ensure that randomization resulted in comparable groups, we tested for differences in means between the two study arms for key program indicators at baseline. Significance was determined using ordinary least squares (OLS) regression adjusted for TA stratification with standard errors (SEs) clustered at the VC level (results in Appendix Table A2).^3^ We find that randomization was successful; mean household characteristics are balanced between the treatment and control groups (no significant differences at the 10 percent level). These results also hold when we tested for differences in VC cluster means (see Table A3).

From the 3,531 households interviewed at baseline, 3,365 households (1,605 treatment and 1,760 control) were interviewed at follow‐up—an attrition rate of 5 percent. The evaluation examined 162 individual and household measures for statistical differences between study arms of the retained households and finds less than 1 percent are different at the 5 percent significance level (Malawi SCTP Evaluation Team, 2015). We also find no evidence of differential attrition from a smaller attrition analysis (Appendix Table A4)^4^ for the subset of key program indicators and all variables used in this analysis.

The analytical sample for this paper includes all 3,365 households that were reinterviewed at follow‐up. There was one respondent for the SWB module per household, typically the main respondent to the household questionnaire, but not necessarily the household head. This household panel, however, includes some households with different respondents to SWB questions in the two survey rounds. Therefore, we also use a smaller subset of these households, the individual panel of 2,919 (1,520 treatment and 1,399 control), consisting of only those households with the same respondent in both surveys.

### Measures

SWB in this analysis is measured using constructs of QoL, future expectations, and relative well‐being. QoL measures are constructed from a series of questions gauging individuals’ perceptions of their life (Douthitt, MacDonald, & Mullis, 1992). This is considered a cognitive process, where a person's judgments are dependent upon a comparison of one's present circumstances with a standard that each individual sets for him or herself (Diener et al., 1985). Therefore, we did not externally impose any reference for comparison so that SWB measures center on a person's own judgments.

To measure the QoL, respondents were asked how much they agree with the following statements, from strongly agree (5) to strongly disagree (1):

1. In most ways my life is close to ideal.
2. The conditions in my life are excellent.
3. I am satisfied with my life.
4. So far I have gotten the important things I want in life.
5. If I could live my life over, I would change almost nothing.
6. I feel positive about my future.
7. I generally feel happy.
8. I am satisfied with my health.

These questions are drawn from the Satisfaction with Life Scale (SWLS) (Diener et al., 1985) and the World Health Organization Quality of Life Scale (WHOQOLS)(WHO, 1998). The first five questions comprise the SWLS, which is narrowly focused on an individual's overall life satisfaction. The SWLS has shown good internal consistency and construct validity (Kobau et al., 2010). The last three questions come from the WHOQOLS and cover positive effect as well as overall QoL. We develop a summary index measure for QoL with a resulting scale that ranges from 8 to 40, higher scores reflecting a greater QoL. It is intended to be a broad measure of QoL incorporating affective and life satisfaction questions because happiness is a complex concept and we want to capture individuals’ perceptions of well‐being across various concepts. Our QoL Scale was not validated in our local context but the internal consistency is respectably high with a Cronbach's alpha score of 0.83 (using both waves of data). Additionally, factor analysis reveals a single construct, consistent with the literature on life satisfaction scales (Frey & Stutzer, 2002). Moreover, results provided in Table A6, 5 demonstrate that program impacts are comparable across individual questions.

The second construct, future expectations, is measured by asking respondents for their perception of how they feel their life will be (either better, same, or worse) in one, two, and three years from now. Binary indicators measure whether individuals feel their life in the future will be better, the same, or worse, compared to what it is now. As compared to life satisfaction, which is an assessment of one's current circumstances, these questions on future well‐being have respondents gauge the unknown future and tap into concepts of expectation and optimism. Psychological theory proposes that as a personality trait, optimism would affect SWB through expectations about the future (Scheier & Carver, 1985). Some literature has found that dispositional optimism correlates well with other measures of SWB such as life satisfaction and positive effect (Lucas, Diener, & Suh, 1996).

This study also solicited caregiver perceptions of their relative well‐being with respect to their societal economic status. Literature has confirmed that income evaluated relative to others has a significant effect on individuals’ perception of well‐being, at least among developed societies (Clark, Frijters, & Shields, 2008). Evidence from developing societies is more inconsistent. For example, Ravallion and Lokshin (2010) find that among the poor in Malawi, SWB is not correlated with mean income in one's neighborhood. However, Fafchamps and Shilpi (2009) find that relative consumption is an important predictor of SWB among the poor, even in isolated villages in Nepal.

We measure relative well‐being using a visual stepladder with six choices, from poor (1) to rich (6). Respondents are asked to place themselves on one of these steps, in addition to their neighbors and friends. We generated two binary variables, one that measures relative well‐being in comparison to friends and the other in comparison to neighbors. The variables measure if individuals perceive themselves to be either the same or better off (compared to worse off) than their friends and neighbors.

Additionally, we tested the relationship between negative shocks and SWB to ensure that our measures are sensitive to negative shocks and respond appropriately. If increases in income increase happiness, shocks that would reduce income, such as the death of an income earner, should analogously have a negative impact on happiness. Respondents were asked about negative shocks that occurred within the previous 12 months. We tested two measures: total number of shocks and an indicator for the death of an income earner. In addition, respondents were asked to assess the likelihood of experiencing negative shocks in the upcoming year: a food shortage and needing financial assistance. We created indicators for whether the respondent believes there is a likely or very likely chance that they will experience each shock in the future.

### Baseline Analysis

Table 2 reports mean values of baseline characteristics and subjective outcomes separately for eligible T and C groups and for the Ineligibles. At baseline, we interviewed 821 ineligible households that were randomly selected from the same VCs. We present baseline mean values for this group of Ineligibles to place our sample in context with the communities they live in and provide evidence that subjective measures correspond to expected objective characteristics.

**Table 2:** Baseline summary statistics for the panel of (eligible) caregivers and the Ineligibles

	Treatment	Control	Ineligibles
	Mean (SD) or %		
**Individual and household characteristics**			
Age	57.9 (19.9)	56.1 (19.6)	47.2 (18.7)
Female	82.8	84.2	64.8
Married	30.0	29.9	68.0
Ever attended school	31.0	32.6	55.1
Chronic illness	46.2	41.1	27.8
Per capita expenditure	45,651 (35,163)	43,369 (31,572)	58,042 (50,132)
Number of persons in household	4.6 (2.3)	4.6 (2.3)	5.0 (2.2)
Number of shocks (last 12 months)	2.6 (1.3)	2.5 (1.3)	2.6 (1.4)
Death in the household (last 12 months)	4.0	3.1	2.2
Believes will have future shocks	54.0	53.9	37.6
**Subjective well‐being outcomes**			
QoL Scale score^a^	17.5 (6.6)	18.1 (6.8)	21.2 (7.5)
**QoL Scale items** b			
In most ways my life is close to ideal	2.0 (1.1)	2.0 (1.2)	2.4 (1.2)
The conditions in my life are excellent	2.1 (1.2)	2.2 (1.3)	2.5 (1.3)
I am satisfied with my life	2.4 (1.3)	2.5 (1.4)	2.9 (1.3)
So far I have gotten the important things I want in life	1.8 (1.1)	1.8 (1.0)	2.1 (1.2)
If I could live my life over, I would change almost nothing	2.3 (1.3)	2.3 (1.4)	2.5 (1.3)
I feel positive about my future	2.2 (1.2)	2.3 (1.2)	2.8 (1.3)
I generally feel happy	2.3 (1.2)	2.4 (1.2)	2.8 (1.3)
I am satisfied with my health	2.5 (1.3)	2.6 (1.4)	3.2 (1.4)
**Future well‐being**			
Better in a year	52.6	53.0	66.8
Better in two years	45.3	47.1	61.0
Better in three years	42.4	46.3	58.5
**Relative well‐being**			
Same or better off than neighbors	43.2	48.6	63.5
Same or better off than friends	48.5	51.3	62.1
**Placement on ladder** c			
Self (1–6)	1.2 (0.5)	1.2 (0.5)	1.6 (0.7)
Neighbors (1–6)	1.9 (0.8)	1.9 (0.9)	1.9 (0.9)
Friends (1–6)	1.9 (1.0)	1.9 (1.0)	2.0 (1.0)
*Observations*	*1,605*	*1,760*	*821*
*Clusters*	*14*	*15*	*29*

*Notes*: No significant differences between T and C (*P*‐value < 0.1) based on *t*‐tests with standard errors clustered at the VC level.

^a^Range of 8 to 40 from the sum of scale item questions (scored 1 to 5).

^b^Scale items from 1 (Strongly disagree) to 5 (Strongly agree).

^c^Ladder from 1 (poor) to 6 (rich).

To ensure that the initial balance achieved still holds for the panel sample used in this analysis, we compared baseline characteristics of caregiver respondents (all outcome and control variables) across treatment and control groups. We tested for differences in means between treatment (T) and control (C) study arms, adjusting for the stratification variable (TA residence) and clustering SEs at the VC level (Table 2). All variables used in this paper are balanced at baseline; we found no statistically significant differences at the 10 percent significance level. In additional tests, we also looked for differences in cluster means (since randomization was done at the cluster level) for all variables in Table 2 and found equivalent results (Table A5).^6^

The baseline characteristics presented in Table 2 show that the vast majority of caregiver respondents are female (over 80 percent) with an average age just below 60. Around a third of the sample has attended school at some point in their life and another third is currently married. In comparison to T and C caregiver samples, the Ineligible group of caregivers looks slightly different. They are younger (average age of 47) and less likely to be female, but are much more likely to be married and to have ever attended school. Since the eligible T and C households are supposed to be the poorest households in the community, ineligible households are wealthier—per capita expenditure is around 30 percent greater. These indicators point to the success of targeting the poorest, labor‐constrained households in these communities.

For future well‐being, the majority of eligible households (53 percent) think that their life will be better in one year. The proportion believing life will be better decreases below 50 percent, however, when respondents think about their life two or three years in the future. A larger percentage of respondents from ineligible households believe life will be better in one year (67 percent) but responses follow the same pattern of decreasing in the more distant future.

For relative well‐being, Table 2 shows the mean values of caregivers’ placements (self, friend, and neighbor) on the economic wealth stepladder and calculated indicators for whether they consider themselves the same or better off than (1) friends and (2) neighbors. At baseline, both T and C respondents consider themselves to be at the bottom. On a scale of 1 (poor) to 6 (rich), respondents have a mean score of 1.2 or “poor.” In comparison, respondents placed both their friends and neighbors almost a step above themselves with mean values around 1.9. This translates into around half of eligible respondents believing they are the same or better off than either their friends or neighbors. Ineligible respondents perceive themselves to be slightly higher up the ladder (1.6), but also place their friends and neighbors at around 2 on the ladder, which might shed some light on how much relative wealth might be a shared community concept.

### Determinants of SWB Measures

We additionally examined individual and household determinants of our SWB measures. In general, positive correlates to SWB include income, self‐perceived good health, and marital status (being married), while female gender and age are more likely to be negative correlates (Diener, Lucas, & Oishi, 2009).

The determinants of SWB are typically modeled empirically as an additive function of the social, economic, and environmental factors involved:

$$SWB_{it}\;=\alpha +\beta_{1}X_{1_{it}}+\beta_{2}X_{2_{it}}+\cdots +\varepsilon_{it},$$

where the error term (*ε_it_*) captures individual differences in reporting (Dolan, Peasgood, & White, 2008). We use this basic model to assess the determinants of SWB at baseline, using three measures that represent each SWB construct: QoL, relative well‐being, and future expectations. The QoL Scale is a continuous variable while binary indicators are used for future well‐being (believe that life will be better in two years) and relative well‐being (believe that they are the same as or better off than neighbors).^7^

Table 3 shows that log per capita expenditure has a significant, positive relationship with QoL scores at baseline. In addition, log per capita expenditure is a strong, positive predictor of future well‐being (*P*‐value < 0.05) but it is not predictive of relative well‐being. Results also indicate that health status and being married have important relationships with SWB. Having a chronic illness is significantly predictive of lower QoL scores by 1.5 points and is associated with being less positive about the future. Being married, on the other hand, is a positive determinant of QoL. Married caregivers have scores 1.2 points higher than non‐married caregivers. Indeed, being married is the only significant determinant across all outcomes.

**Table 3:** Baseline determinants of subjective well‐being among caregivers

	Life satisfaction: Quality of Life Scale	Future well‐being: Life will be better in two years	Relative well‐being: Same or better off than neighbors
Female	−0.08	−0.03	−0.05**
	(0.45)	(0.03)	(0.02)
Age	−0.07**	−0.00*	0.00
	(0.03)	(0.00)	(0.00)
Age squared	0.00	0.00	−0.00
	(0.00)	(0.00)	(0.00)
Ever attended school	−0.47	0.03	0.03*
	(0.32)	(0.02)	(0.02)
Chronic illness	−1.49***	−0.05**	−0.01
	(0.49)	(0.02)	(0.02)
Married	1.17***	0.05**	0.05*
	(0.29)	(0.02)	(0.02)
Log per capita expenditure	1.28***	0.07**	0.02
	(0.33)	(0.02)	(0.02)
Household size	0.24*	0.01	0.02
	(0.12)	(0.01)	(0.01)
Household members 0–5 years	−0.17	−0.01	−0.04**
	(0.20)	(0.02)	(0.02)
Household members 6–11 years	−0.34*	−0.00	−0.02
	(0.17)	(0.01)	(0.02)
Household members 12–17 years	−0.03	0.02	−0.02
	(0.14)	(0.01)	(0.02)
Household members 65 and over	−0.36	−0.04**	−0.01
	(0.19)*	(0.01)	(0.02)
Treatment	−0.42	−0.01	−0.06
	(0.82)	(0.05)	(0.04)
Constant	5.93	0.04	0.29
	(3.82)	(0.26)	(0.19)
*Observations*	3,365	3,365	3,365
*R^2^*	0.06	0.07	0.04

*Notes*: ^*^ *P* < 0.1; ^**^ *P* < 0.05; ^***^ *P* < 0.01. Estimates are from OLS regressions with robust standard errors in parentheses, clustered at the VC level. Regressions also include TA residence dummies to account for stratification in the study design.

Other significant determinants of QoL at baseline include a caregiver's age (negatively associated) and household composition variables (both positive and negative). Future and relative well‐being display a similar pattern of relationships to determinants but compared to the QoL Scale, they have fewer significant coefficients. Notably, female gender is only predictive of relative well‐being (a negative association). The heavy saturation of female caregivers in the sample, however, means there is not much gender variation to test.

Overall, determinant analysis reveals that the QoL Scale is more strongly predicted by individual and household variables than both future and relative well‐being. Consumption, poor health, and being married are the most significant predictors of SWB outcomes and have expected relationships, in line with the literature.

## METHODS

To estimate the average treatment effect of the cash transfer on our outcomes we use panel data and a Differences‐in‐differences (DD) regression model. Equation (1) is our main empirical specification where *Y_it_* is an individual, time‐specific measure of SWB, *T_i_P_t_* is an indicator for cash transfer receipt in the second wave and represents the DD treatment effect, which is the product of treatment status (*T_i_*) and the post period (*P_t_*).

(1) $$Y_{it}=\;\alpha +\beta_{1}\left(T_{i}P_{t}\right)+\beta_{2}T_{i}+\beta_{3}P_{t}+\beta_{4}X_{it}+e_{it}$$

We first estimate a basic model without controls and then adjust for a set of baseline individual and household covariates (*X_it_*). Dummy variables for TA residence, however, are included in all models to account for randomized sampling procedures stratified by TA. The adjustments in *X_it_* consist of individual correlates of SWB including age, age‐squared, gender, marital status, education, and chronic health issues (Dolan, Peasgood, & White, 2008; Weimann, Knabe, & Schöb, 2015). Household correlates controlled for include household size and total members in age groups (0 to 5, 6 to 11, 12 to 17, 18 to 65, and 65‐plus).

We then provide some additional analysis with sensitivity and robustness checks, and conclude with an examination of potential pathways through which the Malawi SCTP could affect SWB. We assess six objective program impacts that improve household welfare and could have been affected within the study time frame: number of meals per day, consumption (total and food), ability to respond to shocks, and likelihood of future shocks. Number of meals is operationalized with an indicator for whether the household eats two or more meals per day and consumption variables are operationalized as (1) total log per capita expenditure and (2) log per capita food expenditure. Ability to respond to shocks is an indicator for whether they had a negative shock and used their own money to respond, and likelihood of future shock is whether they believed they would have a financial or food shock in the next 12 months. Using the above regression specifications, we first estimate the treatment effect on objective mediators and then reestimate our outcome model including each mediator independently. We compare the coefficient of the treatment effect $\left(T_{i}P_{t}\right)$ to the total effects to determine whether there is any attenuation of the program impact after taking into account the mediator. Any observed attenuation is evidence the program indirectly works through that pathway to impact SWB.

SEs for all regressions are clustered at the VC level, the level of random assignment to treatment. Due to our study design, we have a relatively small number of clusters (29 VCs) and consequently regular cluster adjusted SEs may be too small leading to an over‐rejection of the null or no effect. We therefore report both cluster‐robust SEs (CRSE) and *P*‐values based on the wild bootstrap method (Cameron, Gelbach, & Miller, 2008), which adjust for the small number of clusters.

## RESULTS

**Figure 1:**
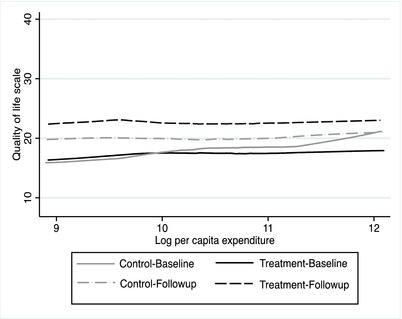
QoL Scale Over Household Consumption by Study Arm and Wave. *Note*: Lowess graph showing the change in QoL scores over time for each study arm.

Since the expectation is that a positive income shock can increase happiness, it is important to understand how consumption impacts SWB. Figure 1 graphically represents the relationship between consumption (per capita expenditure in logarithms) and QoL scores (range of 8 to 40) using a local linear regression (lowess) model.

As mentioned earlier, consumption is much lower at follow‐up due to data collection occurring during the lean season. Therefore, the control group is the key to our estimation strategy as it accounts for this seasonality. Overall, the relationship is mostly flat across expenditure levels for both T and C groups. At follow‐up, however, scores increase horizontally for the T group but also for C at lower levels of consumption. Nevertheless, T scores are clearly higher than C scores across all levels of consumption at follow‐up.

### Impact of Malawi SCTP on SWB

Estimates of the average treatment effects of the Malawi SCTP on caregiver SWB are displayed in Table 4. All estimates are from our main DD specification, but for greater transparency of our results, we also provide estimates from single‐differences and fixed effects specifications in the Appendix (Table A7).^8^ The first column for each outcome in Table 4 is the unadjusted estimate (but which also includes the stratification variables) for the full household panel. The adjusted estimates in the second column add individual and household controls plus a full set of treatment‐covariate interactions. The last columns are the adjusted estimates from the individual panel.

**Table 4:** Estimates of the average treatment effect of Malawi's SCTP on measures of subjective well‐being

	Quality of Life Scale			Life will be better in two years			Same or better off than neighbors		
	(1)	(2)	(3)	(4)	(5)	(6)	(7)	(8)	(9)
Treatment effect (DD)	3.29***	3.18***	3.38***	0.19***	0.19***	0.20***	0.11	0.11	0.12*
	(0.79)	(0.80)	(0.85)	(0.06)	(0.06)	(0.06)	(0.07)	(0.07)	(0.07)
*Wild bootstrap P‐value*	0.000	0.000	0.001	0.003	0.007	0.004	0.112	0.119	0.105
Treatment	−0.55	0.01	−0.13	−0.01	0.39	0.22	−0.06	0.25	0.40
	(0.84)	(5.00)	(5.56)	(0.05)	(0.35)	(0.40)	(0.04)	(0.25)	(0.28)
Time	1.74***	1.78***	1.64***	0.05*	0.05	0.04	−0.06	−0.06	−0.06
	(0.54)	(0.54)	(0.57)	(0.03)	(0.03)	(0.03)	(0.05)	(0.05)	(0.05)
Demographic controls		X	X		X	X		X	X
Household controls		X	X		X	X		X	X
Full set of treatment‐covariate interactions		X	X		X	X		X	X
Individual panel			X			X			X
*Control mean*	18.10	18.10	18.10	0.47	0.47	0.48	0.49	0.49	0.48
*Observations*	6,896	6,733	5,838	6,370	6,207	5,374	6,884	6,721	5,826
*R^2^*	0.10	0.14	0.13	0.04	0.10	0.10	0.01	0.03	0.03

*Notes*: ^*^ *P* < 0.1; ^***^ *P* < 0.01. Robust standard errors in parentheses clustered at the VC level; Wild *t*‐bootstrap *P*‐value for DD coefficient (1,000 reps, H_0_ = 0) shown in underneath program effect. Controls include Demographics (female, age, age squared, ever attended school, chronic illness, married) and Household characteristics (baseline values of log per capita expenditure, household size, total age group categories—0 to 5, 6 to 11, 12 to 17, 65‐plus). Dummies for TA residence are included in all models as this was the level of stratification in randomization. Control mean is the dependent variable's baseline mean in the control group.

The unadjusted results indicate that there is a significant treatment effect for two outcomes, the QoL Scale (column 1) and future well‐being (column 4). Caregivers in treatment households score 3.3 points higher on the QoL Scale, which is 19 percent of the mean baseline score and translates to 0.48 of an SD increase. Additionally, these caregivers are 19 percentage points more likely to believe in a better future over their counterparts in the control group. For both of these outcomes, there is no difference between the significance of CRSEs and the wild *t*‐bootstrap test; both provide *P*‐values < 0.01.

Compared to the unadjusted estimates, the addition of covariates only slightly changes treatment effect point estimates. For the QoL Scale, the program impact (columns 2 and 3), is still strongly significant (*P*‐value < 0.01) across both the CRSE and wild bootstrap test for each model. The magnitude of the point estimate is slightly reduced after the inclusion of controls in the household panel (column 2) but point estimates are larger for the individual panel (column 3). Effect sizes remain around 0.5 SD, which is larger than the impacts from the GiveDirectly study that reported a 0.26 SD increase in an index measure of psychological well‐being, including a 0.16 SD effect on happiness and a 0.17 SD effect on life satisfaction (Haushofer & Shapiro, 2016). Time also has a significant effect on QoL scores, an impact of about 1.6 to 1.8 points, consistent with the upward shift in the QoL curve in Figure 1 in the control group. While there is no significant imbalance of scores at baseline, means are not exactly even between T and C (17.5 vs. 18.2, respectively), so while the treatment impact is still significant in the single‐difference model in Appendix Table A5,^9^ point estimates are slightly smaller compared to our main results. Thus, controlling for baseline differences and time trends in our DD model is important and helps to increase internal validity.

Columns 5 and 6 in Table 4 show that treatment effects for “Life will be better in two years” are the same as the unadjusted point estimate. Beneficiary caregivers are 19 or 20 percentage points more likely to believe in a better future (*P*‐value < 0.01). Lastly, while results are positive for relative well‐being across specifications, the only significant estimate is for the individual panel at the 10 percent level (column 9)—caregivers are 12 percentage points more likely to believe that they are the same or better off than their neighbors.

Overall, Table 4 shows that the impact of the cash transfer is robust across specifications and point estimates are marginally higher for the individual panel. Furthermore, the program impact for QoL and future well‐being displays strong significance (*P*‐value < 0.01) using either the CRSEs or the wild bootstrap test, signifying that regression SEs are unbiased. Given the robustness of the treatment effect across specifications, we focus on the individual panel sample for our subsequent analyses as this sample reduces concerns about different reporting scales across individuals over time.

### Sensitivity Analysis

Our analysis so far has demonstrated high internal validity. Estimates of the treatment effect are consistent and robust across specifications. Internal validity, however, is also dependent on the ability of the measure to correctly represent the concept it defines. In particular, construct validity is a relevant concern for the QoL Scale, as it is generated from a series of response items, some of which cover positive effect as well as life satisfaction. We first tested each QoL item individually for a program effect, and except for item 5 (“Would you relive your life but change almost nothing?”), we find significant effects of around half a point for each measure. Results can be found in the Appendix (Table A6).^10^

Additionally, as a sensitivity analysis to test the construct validity of the QoL Scale, we examine whether the scale predicts negative shocks in the expected negative direction (opposite to a positive income shock) to confirm that it incorporates appropriate emotional effect in response to one's experiences. Using our preferred model specification, we test the effect of three measures of negative shocks on QoL including the number of shocks and household death (both in the previous 12 months), and the anticipation of a future shock (either financial or food) in the next 12 months. We also include the treatment effect as a control in a second model to further see whether the cash transfer is protective of life satisfaction above these negative shocks.

Shocks could arguably be endogenous to treatment and we thus depart from causal analysis to validate this construct. In Table 2, however, we show that these shocks are balanced at baseline. At follow‐up, the total number of shocks in the previous year decreased from a mean of 2.5 to fewer than 2 for both groups, but the percentage of the sample that experienced a death remained constant at around 3 percent. Additionally, both groups were less likely to believe they would suffer from future shocks at follow‐up (34 percent for T and 44 percent for C), declining from 53 percent at baseline.

Results in Table 5 uphold the construct validity of the QoL Scale. They show that each shock has the expected negative relationship with QoL scores, although only number of shocks and belief in future shocks are significant. Each additional shock a household experienced in the previous 12 months decreases QoL scores by almost one point, significant at the 1 percent level. Likewise, believing one will have future shocks decreases scores by 2.6 points, also significant at the 1 percent level. With the addition of the DD indicator, both total shocks and future negative shocks still have a significant impact on QoL scores and point estimates are on the same order of magnitude. Moreover, treatment effects in columns 2, 4, and 6 are nearly the same as those in Table 4, further validating the robustness of the program impact on QoL for beneficiary households. Negative shocks and the positive income shock, therefore, appear to be orthogonal to each other and life satisfaction is an experience that responds to multiple external events at the same time.

**Table 5:** Sensitivity analysis using relationships between negative shocks (real and anticipated) and the Quality of Life Scale

Dependent variable = Quality of Life Scale						
	Number of shocks (last 12 months)		Death in household (last 12 months)		Believes will have future shocks (next 12 months)	
Type of shock	(1)	(2)	(3)	(4)	(5)	(6)
Effect of Shock	−0.90***	−0.90***	−0.73	−0.59	−2.60***	−2.49***
	(0.11)	(0.11)	(0.55)	(0.57)	(0.31)	(0.31)
*Wild bootstrap P‐value*	0.000	0.000	0.187	0.299	0.000	0.000
Treatment effect (DD)		3.40***		3.37***		3.04***
		(0.74)		(0.85)		(0.82)
*Observations*	5,838	5,838	5,838	5,838	5,838	5,838
*R^2^*	0.146	0.161	0.118	0.133	0.152	0.165

*Notes*: ^***^ *P* < 0.01. Estimates are for the individual panel. Robust standard errors in parentheses clustered at the VC level; Wild *t*‐bootstrap *P*‐value (1,000 reps, H_0_ = 0) for negative shock shown below shock coefficient. Controls include Demographics (female, age, age squared, ever attended school, chronic illness, married) and Household characteristics (baseline values of household size, total age group categories—0 to 5, 6 to 11, 12 to 17, 65‐plus—and dummies for TA residence). Models also include treatment and time dummies and a full set of control–treatment interactions.

### Possible Mechanisms

While our analysis points to the clear conclusion that Malawi's SCTP has a positive impact on caregiver SWB, there could be a variety of reasons for this result. Increased expenditures on food and non‐food items is one likely mechanism. As food shares constitute upwards of 80 percent of our study sample's expenditure, increased food consumption might have an important impact on QoL by reducing hunger and worry over food. Expenditure on non‐food items such as clothes, toiletries, and housing improvements can also affect well‐being by improving individuals’ self‐esteem and feeling of pride or dignity (Bastagli et al., 2016). For example, purchasing uniforms may improve caregivers’ sense of dignity because it allows them to send their children to school. Lastly, the program may trigger increases in well‐being as recipients become more self‐reliant and the cash allows them to plan for the future (Bastagli et al., 2016).

We extend analysis in this last section to examine whether other program impacts might be mediating the total treatment effect on SWB. Given that the immediate impacts of the cash transfer programs mostly go towards alleviating chronic issues of hunger, purchasing essential non‐food goods, and managing shocks—we focus on measures of food security, consumption, and self‐reliance to respond to shocks. Table 6 shows program impacts for five outcomes (coded in the direction we anticipate the mechanism working): whether households eat over one meal per day, log per capita expenditure, log per capita food expenditure, whether they foresee a future without a financial or food shock (in the next 12 months), and whether households relied on their own funds when they had a negative shock.^11^

**Table 6:** Impact of Malawi's SCTP on household food security, consumption, and ability to respond to shocks

	Over one meal/day	Log per capita expenditure	Log per capita food expenditure	Not likely to have future financial or food shock	Relied on savings or SCT
Treatment effect (DD)	0.09**	0.20**	0.19**	0.14*	0.31***
	(0.04)	(0.07)	(0.09)	(0.07)	(0.09)
*Wild bootstrap P‐value*	0.017	0.017	0.043	0.075	0.009
*Observations*	5,837	5,838	5,836	5,838	5,068
*R^2^*	0.064	0.322	0.268	0.045	0.103

*Notes*: ^*^ *P* < 0.1; ^**^ *P* < 0.05; ^***^ *P* < 0.01. Estimates are for the individual panel. Robust standard errors in parentheses clustered at the VC level; Wild *t*‐bootstrap *P*‐value (1,000 reps, H_0_ = 0) for program effect shown below DD coefficient. Controls include Demographics (female, age, age squared, ever attended school, chronic illness, married) and Household characteristics (baseline values of household size, total age group categories—0 to 5, 6 to 11, 12 to 17, 65‐plus—and dummies for TA residence). Models also include treatment and time dummies and a full set of control–treatment interactions.

Results in Table 6 show significant, positive treatment effects for each of these measures.

The program increases both per capita total and food expenditure, helps households ensure that they eat more than one meal per day, improves perceptions about future shocks, and increases their reliance on their own money to deal with negative shocks.

To test whether these outcomes help explain some or all of the total treatment effect of the program on SWB, we employ a brief mediation analysis by modeling the impact of each mediator on the treatment effect (for a review of the causal mediation framework to identify direct and indirect effects of policy interventions, see Keele, Tingley, & Yamamoto, 2015). For this step, we reestimate our outcome models using our preferred specification but with the addition of each objective measure (included separately). Regressions also include baseline values of the objective measure to control for any confounding between the mediator and treatment, a necessary step to justify the sequential ignorability assumption because mediators were not randomized (Keele, Tingley, & Yamamoto, 2015).

Table 7 shows that these objective measures do little to mediate the treatment effect (first row) for either the QoL Scale or future well‐being. Treatment effects for the QoL Scale are only marginally lower than the total effect from Table 4 (3.38 points) and results are still significant at the 1 percent level. The same is true for future well‐being, none of the objective outcomes diminishes the direct treatment effect from Table 4 (19 pp).

**Table 7:** Analysis of potential mechanisms to explain subjective well‐being results

Mediator:	Over one meal/day	Log per capita expenditure	Log per capita food expenditure	Not likely to have future financial or food shock	Rely on own money
	(1)	(2)	(3)	(4)	(6)
*Panel A. Dependent variable: Quality of Life Scale*					
Treatment effect (DD)	3.23***	3.17***	3.20***	3.05***	3.35***
	(0.84)	(0.85)	(0.84)	(0.82)	(0.92)
*Wild bootstrap P‐value*	0.001	0.001	0.001	0.002	0.002
Mediator	1.64***	1.06***	0.93***	2.42***	−0.11
	(0.44)	(0.30)	(0.25)	(0.30)	(0.47)
*Wild bootstrap P‐value*	0.000	0.001	0.002	0.002	0.002
*Observations*	5,837	5,838	5,836	5,838	5,068
*R^2^*	0.136	0.136	0.135	0.162	0.112
*Panel B. Dependent variable: Life will be better in two years*					
Treatment effect (DD)	0.19***	0.19***	0.19***	0.19**	0.20***
	(0.06)	(0.07)	(0.07)	(0.07)	(0.06)
*Wild bootstrap P‐value*	0.006	0.013	0.012	0.015	0.003
Mediator	0.12***	0.07***	0.06***	0.14***	0.04
	(0.03)	(0.02)	(0.02)	(0.02)	(0.03)
*Wild bootstrap P‐value*	0.002	0.013	0.005	0.015	0.003
*Observations*	5,373	5,374	5,372	5,374	4,669
*R^2^*	0.102	0.098	0.098	0.113	0.095

*Notes*: ^**^ *P* < 0.05; ^***^ *P* < 0.01. Each column within a panel reports results from a separate regression using the individual caregiver panel. Robust standard errors in parentheses clustered at the VC level; Wild *t*‐bootstrap *P*‐value (1,000 reps, H_0_ = 0) shown below standard errors. Controls include Demographics (female, age, age squared, ever attended school, chronic illness, married) and Household characteristics (baseline values of the objective outcome controlled for, household size, total age group categories—0 to 5, 6 to 11, 12 to 17, 65‐plus—and dummies for TA residence). Models also include treatment and time dummies and a full set of control–treatment interactions.

Similar to findings on the relationship between QoL scores and per capita consumption (Figure 1), one possibility that might explain results in Table 7 is that at least in the short‐term, SWB in the treatment group improved uniformly across household conditions. Thus, cash transfer receipt would itself be enough to improve happiness at an even rate across caregivers likely because it improves life circumstances in at least some significant way for all beneficiaries. The evidence from Table 6 lends support to this explanation as it shows each measure has a significant and positive relationship with SWB.

## DISCUSSION

This study reveals that in just about a year's time, Malawi's cash transfer can have a profound effect on the SWB of caregivers in beneficiary households. We find a strong, positive impact of the income shock on individuals’ QoL and perception of future well‐being but do not find a similar strong impact on their perception of relative well‐being. This finding lines up with evidence of positive impacts on objective measures of well‐being at the household and individual levels. For example, the cash importantly helps to increase food expenditures and the number of meals eaten compared to the control group, showing that the program was particularly protective for households during the lean season when the survey was conducted. However, we find no evidence that these increases in objective well‐being measures explain the direct program effect on subjective outcomes.

This quantitative evidence from Malawi is also substantiated by qualitative evidence from in‐depth caregiver interviews collected at follow‐up, which also give some insight on how the program has changed their lives (Malawi SCTP Evaluation Team, 2015). Caregivers in beneficiary households describe how the cash has been crucial for them to afford to eat regular meals, make home improvements, buy livestock, and send their children to school. Many of their stresses are alleviated, making them happier. Additionally, caregivers admit that they are hopeful for the future. General feelings are that they believe their lives will continue to get better and their children's future will be more promising as they are able to continue with their education. In general, the qualitative transcripts clearly indicate that the mental/emotional impacts of having a secure source of income is extremely important and helps explain why the objective measures do not explain the increases in SWB brought about by the SCTP.

In addition to corroborating evidence from the objective measures and qualitative data, we find that the results of the cash transfer program on SWB are robust across all specifications and models. Moreover, results show that negative and positive shocks together can have strong, independent impacts on QoL, possibly reflecting how positive and negative psychological states can exist simultaneously (Diener, Larsen, & Emmons, 1984; Watson, Clark, & Carey, 1988). Literature has even found that in times of severe stress such as the death of a family member, co‐occurrence of positive and negative psychological states is common and is part of the coping process (Folkman, 1997).

Interestingly, while results are strongly positive for measures of QoL and future well‐being, we find no impact on relative well‐being. According to the literature, people's happiness is judged relative to an internal reference point, which is determined by their past experiences and environments. Therefore, the perception of low relative economic standing in a community reflects lower happiness because, compared to others, there is potential to be happier. As we reported earlier, for most households transfer size as a share of pre‐program household consumption is lower than the generally accepted 20 percent threshold. It might be that this modest increase in income is not enough for households to consume as much as their friends and neighbors and so, relative to their community, they are still worse off. Therefore, the absolute income effect is likely driving the positive results we see for QoL and future well‐being. The null effect seems to align with prior work in Malawi that found no impact of income on relative well‐being among the poorest communities (Ravallion & Lokshin, 2010).

While all of these positive impacts would in most respects appear to be good news, there is a concern amongst some that such programs could result in “leapfrogging.” In the context of cash transfer programs, leapfrogging refers to a situation when program beneficiaries quickly move to a higher standard of living that leaves behind other community members who were nearly as poor but did not receive the program (Ellis, 2012). In this situation, feelings of unfairness and bitterness could arise and lead to lowered social cohesion within the community. This could help explain the negative externality of lowered SWB for neighbors of GiveDirectly recipients since transfers were much larger (up to two years of per capita expenditure) over a nine‐month period (Haushofer & Shapiro, 2016). The Malawi SCTP, like most national cash transfer programs in SSA, distributes smaller transfers, and only to a small percentage of the community, so leapfrogging is likely less of a concern. The relative well‐being results also support this since beneficiary caregivers still do not rate themselves as better off than their neighbors or friends.

### Limitations

Despite the strength of our results, the positive time trend is an anomaly. It is unclear why control households report higher life satisfaction and future outlooks at the second wave. There was no concurrent rise in external economic circumstances, and in fact follow‐up data collection occurred during the lean season when consumption was much lower for all households, a decline of around 25 percent from baseline (Malawi SCTP Evaluation Team, 2015). While it was the lean season, it was also the rainy season during follow‐up data collection, and a possible connection could exist between the rains and SWB if the rains signal that the growing season is under way and bounty is to come. However, some recent literature has rejected the use of intrapersonal comparisons (Weimann, Knabe, & Schöb, 2015). According to Rayo and Becker (2007), people develop internal references in accordance with their life circumstances as an evolutionary response in order to sustain a minimum level of satisfaction. Therefore, individuals’ criteria for a satisfactory life can change over time depending on context. While this could create noise in our estimates, the large sample size and experimental design help guard against this threat to our results—the noise would randomly be assigned. Even withstanding this interference, we are not making conclusions about the intrinsic value of measures, but, instead, are concerned with how an exogenous income shock affects trends over time. Nevertheless, we interpret our findings as short‐term impacts since we do not have the ability to make inferences on habituation over multiple time points.

Individual heterogeneities could also present a problem when making interpersonal comparisons. However, we find trivial differences between the household and individual panels suggesting that personality biases are not a problem; a result that is consistent with the literature (Beegle, Himelein, & Ravallion, 2012). On the other hand, we cannot distinguish whether our positive impacts reflect a movement along the same SWB function or an entire shift of the function due to participation in the program. The short time frame of the study would suggest that we have measured movements along the same function.

## CONCLUSION

This study shows that even small income increases are immensely valuable to the very poor. Caregivers use the money to improve their families’ livelihoods, ensuring provision of their basic needs including food, shelter, and clothing. The reduction of these daily stresses makes caregivers happier about their current situations and gives them hope that the future will continue to improve. Future research will be needed to understand if the absolute income effect will continue to have an impact on SWB in Malawi or if happiness flattens out as people adapt to their new circumstances. In Kenya, Haushofer, Reisinger, and Shapiro (2015) find some evidence for hedonic adaptation in the GiveDirectly study as the psychosocial effects of the transfers (positive for the recipients and negative spillover effects for neighbors) diminished after the study ended. In Malawi, however, the more consistent, long‐term transfers (common across other SSA cash transfer programs) compared to the large, short‐term ones from GiveDirectly's program could be an important distinction and lead to different long‐term effects.

Cash transfers and other poverty alleviation program evaluations should continue to include SWB metrics to add to this evidence base. The use of self‐reported well‐being captures a more holistic picture of well‐being than would reliance only on objective measures and helps us answer questions about whether greater SWB can influence enhanced decisionmaking among the poor. With the growth of cash transfer programs across Africa, it will be important to understand whether there is an association between growth in these metrics and successful transition out of the poverty cycle. This critical knowledge can be used to enhance the effectiveness of social protection policy for the poor across Africa.

## Footnotes

1 All appendices are available at the end of this article as it appears in JPAM online. Go to the publisher's website and use the search engine to locate the article at http://onlinelibrary.wiley.com.

2 All appendices are available at the end of this article as it appears in JPAM online. Go to the publisher's website and use the search engine to locate the article at http://onlinelibrary.wiley.com.

3 All appendices are available at the end of this article as it appears in JPAM online. Go to the publisher's website and use the search engine to locate the article at http://onlinelibrary.wiley.com.

4 All appendices are available at the end of this article as it appears in JPAM online. Go to the publisher's website and use the search engine to locate the article at http://onlinelibrary.wiley.com.

5 All appendices are available at the end of this article as it appears in JPAM online. Go to the publisher's website and use the search engine to locate the article at http://onlinelibrary.wiley.com.

6 All appendices are available at the end of this article as it appears in JPAM online. Go to the publisher's website and use the search engine to locate the article at http://onlinelibrary.wiley.com.

7 We include only these measures throughout our analysis for sake of brevity as we find the same pattern of results for other outcomes in each of these areas (see Appendix Table A6). All appendices are available at the end of this article as it appears in JPAM online. Go to the publisher's website and use the search engine to locate the article at http://onlinelibrary.wiley.com.

8 All appendices are available at the end of this article as it appears in JPAM online. Go to the publisher's website and use the search engine to locate the article at http://onlinelibrary.wiley.com.

9 All appendices are available at the end of this article as it appears in JPAM online. Go to the publisher's website and use the search engine to locate the article at http://onlinelibrary.wiley.com.

10 All appendices are available at the end of this article as it appears in JPAM online. Go to the publisher's website and use the search engine to locate the article at http://onlinelibrary.wiley.com.

11 Additionally, the follow‐up evaluation report examines program impacts for a larger set of individual and household outcomes. Notable results include child schooling improvements including increased enrollments (12 pp) and decreased dropouts (−4 pp) as the cash is used to help meet the costs of sending children to school. Moreover, households used the money to increase agricultural productivity, purchase livestock and agricultural assets, and increase crop production (Malawi SCTP Evaluation Team, 2015).

## Appendix

We also estimated treatment impacts using two additional models: a singles differences model (equation (A1)) and a fixed effects model (equation (A2)). Because we have a randomized study with baseline balance we can estimate the effect of the treatment using only follow‐up data and single differences. Table A6 shows coefficient estimates from both unadjusted and adjusted models in the first two columns under each dependent variable. Since randomization was stratified, the unadjusted model includes dummies for TA residence, the level of stratification. The last column under each dependent variable shows the fixed effects model estimate. Individual fixed effects allow us to control for additional characteristics that could affect SWB responses but are unobserved, such as personality and heterogeneous reporting scales.

**Table A1:** Mean caregiver characteristics across SSA social cash transfer programs

	Malawi SCTP	Kenya CT‐OVC	Zimbabwe HSCT	Zambia SCT	Ghana LEAP
Mean age	58	63	60	59	60
Female	84	65	68	60	61
Per capita expenditure per day (USD)	0.34	0.60	0.85	0.43	1.00

*Source*: Authors’ own calculations. Data not publicly available.

**Table A2:** Mean values of key indicators at baseline by treatment status (household‐level means)

	Treatment	Control	Difference (T–C)	*P*‐value
Head female (%)	82.4	83.6	−1.2	0.46
Head age (mean)	58.3	56.3	2.0	0.29
Head ever attended school (%)	33.3	32.0	1.3	0.35
Head literate (%)	19.4	20.7	−1.3	0.91
Head widow (%)	43.9	41.3	2.6	0.51
Head never married (%)	2.7	3.0	−0.3	0.99
Number of persons in household (mean)	4.5	4.6	−0.1	0.99
Per capita expenditure (mean MWK^a^)	46,465	43,780	2,685	0.44
Expenditure per cap < ultra‐poverty line (%)	72.9	74.7	−1.8	0.79
Eat only one meal/day (%)	22.1	20.3	1.8	0.41
Cultivate land (%)	95.7	95.7	0.0	0.95
Sell crops (%)	21.7	21.3	0.4	0.97
Own an enterprise (%)	26.0	23.5	2.5	0.36
Work ganyu labor (%)	57.5	59.5	−2.0	0.66
Work wage labor (%)	4.4	5.7	−1.3	0.40
*Observations*	*1,678*	*1,853*		
*Clusters*	*14*	*15*		

*Note: P*‐values are based on tests for the equality of means across groups, adjusted for TA stratification with standard errors clustered at the VC level.

^a^MWK, Malawi Kwacha.

**Table A3:** Mean values of key indicators at baseline by treatment status (cluster means)

	Treatment	Control	Difference (T–C)	*P*‐value
	VC cluster mean or %			
Head female (%)	82.0	84.0	−2.0	0.33
Head age (mean)	58.7	56.4	2.3	0.22
Head ever attended school (%)	34.7	31.7	3.0	0.29
Head literate (%)	20.4	20.0	0.4	0.82
Head widow (%)	44.7	42.5	2.2	0.57
Head never married (%)	2.7	2.7	0.0	0.99
Number of persons in household (mean)	4.5	4.6	−0.1	0.73
Per capita expenditure (mean MWK^a^)	47,440	43,131	4,309	0.19
Expenditure per cap < ultra‐poverty line (%)	70.8	75.2	−4.4	0.23
Eat only one meal/day (%)	22.6	21.0	1.6	0.43
Cultivate land (%)	95.5	95.6	−0.1	0.98
Sell crops (%)	22.1	20.6	0.5	0.73
Own an enterprise (%)	26.3	23.5	2.8	0.38
Work ganyu labor (%)	56.5	59.8	−3.3	0.49
Work wage labor (%)	4.8	5.3	−0.5	0.63
*Observations*	*1,678*	*1,853*		
*Clusters*	*14*	*15*		

*Note: P*‐values are based on tests for the equality of village cluster (VC) means across groups, adjusted for TA stratification.

^a^MWK, Malawi Kwacha.

**Table A4:** Selective attrition analysis

	Treatment			Control			Difference	
	Attritors	Non‐attritors	*P*‐value	Attritors	Non‐attritors	*P*‐value	col 1–col 4	*P*‐value
	(1)	(2)	(3)	(4)	(5)	(6)	(7)	(8)
**Household key program indicators**								
Head female	0.79	0.83	0.43	0.69	0.84	0.01	−0.09	0.19
Head age	61.19	58.20	0.43	54.48	56.43	0.56	−6.70	0.26
Head ever attended school	0.30	0.33	0.66	0.35	0.32	0.62	0.05	0.63
Head literate	0.21	0.19	0.72	0.29	0.20	0.30	0.07	0.48
Head widow	0.50	0.44	0.49	0.42	0.41	0.94	−0.08	0.50
Head never married	0.01	0.03	0.38	0.04	0.03	0.33	0.03	0.18
Number of persons in household	3.29	4.58	0.00	4.00	4.61	0.02	0.71	0.16
Death in the household (last 12 months)	0.04	0.04	0.92	0.04	0.03	0.56	0.00	0.97
Believes will have future shocks	0.59	0.54	0.54	0.55	0.54	0.87	−0.04	0.70
Number of shocks (last 12 months)	2.47	2.58	0.57	2.63	2.53	0.48	0.15	0.65
Per capita expenditure (MWK^a^)	65,148.46	45,651.30	0.01	51,728.90	43,369.08	0.24	−13,419.56	0.18
Expenditure per cap < poverty line	0.80	0.90	0.08	0.89	0.92	0.54	0.09	0.20
Eat only one meal/day	0.20	0.22	0.70	0.24	0.20	0.32	0.04	0.53
Cultivate crops	0.89	0.96	0.27	0.95	0.96	0.40	0.06	0.41
Sell crops	0.16	0.22	0.15	0.26	0.21	0.39	0.09	0.11
Owns enterprise	0.19	0.26	0.41	0.22	0.24	0.75	0.03	0.71
Ganyu employment	0.46	0.58	0.17	0.48	0.60	0.15	0.03	0.83
Wage employment	0.03	0.04	0.14	0.08	0.06	0.45	0.05	0.21
**Caregiver characteristics**								
Female	0.80	0.83	0.49	0.69	0.84	0.02	−0.11	0.10
Age	61.14	57.89	0.39	54.53	56.17	0.62	−6.62	0.27
Married	0.24	0.30	0.42	0.27	0.30	0.63	0.03	0.74
Ever attended school	0.29	0.31	0.72	0.35	0.33	0.72	0.07	0.53
Chronic illness	0.54	0.46	0.22	0.49	0.41	0.13	−0.05	0.64
**Subjective well‐being outcomes**								
Quality of Life Scale	15.81	17.54	0.06	18.98	18.11	0.43	3.16	0.08
Life will be better in a year	0.56	0.53	0.61	0.58	0.53	0.24	0.03	0.77
Life will be better in two years	0.39	0.45	0.27	0.43	0.47	0.48	0.04	0.62
Life will be better in three years	0.36	0.42	0.33	0.42	0.46	0.47	0.06	0.58
Relative wealth: same or better off than neighbors	0.46	0.43	0.56	0.47	0.49	0.83	0.02	0.86
Relative wealth: same or better off than friends	0.53	0.48	0.33	0.48	0.51	0.60	−0.05	0.52

*Notes*: Overall *N* for control is 1,853 (In study/non‐attritors = 1,762; Attritors = 91). Overall *N* for treated is 1,678 (In study/non‐attritors = 1,608; Attritors = 70). *t*‐Tests based on standard errors clustered at the VC level.

^a^MWK, Malawi Kwacha.

**Table A5:** Baseline summary statistics for the panel of eligible caregivers and the Ineligibles

	Treatment (T)	Control (C)	Ineligibles	*P*‐value (T–C)
	VC cluster mean (SD) or %			
**Individual and household characteristics**				
Age	58.4 (5.7)	56.2 (5.1)	47.3 (5.4)	0.21
Female	82.2	83.9	64.2	0.38
Married	29.2	28.7	68.1	0.81
Ever attended school	31.7	32.6	55.5	0.97
Chronic illness	47.1	41.3	27.5	0.17
Per capita expenditure	47.367 (8,753)	43,111 (10,008)	58,071 (14,899)	0.24
Number of persons in household	4.5 (0.5)	4.6 (0.7)	5.0 (0.5)	0.71
Number of shocks (last 12 months)	2.6 (0.5)	2.5 (0.6)	2.6 (0.6)	0.84
Death in the household (last 12 months)	4.3	3.3	2.1	0.25
Believes will have future shocks	54.3	54.6	37.1	0.95
**Subjective well‐being outcomes**				
QoL Scale score^a^	17.4 (2.3)	18.0 (2.6)	21.4 (3.0)	0.55
**QoL Scale items** b				
In most ways my life is close to ideal	2.0 (0.3)	2.0 (0.3)	2.4 (0.4)	0.73
The conditions in my life are excellent	2.1 (0.3)	2.2 (0.4)	2.5 (0.5)	0.43
I am satisfied with my life	2.4 (0.3)	2.5 (0.4)	2.9 (0.4)	0.45
So far I have gotten the important things I want in life	1.8 (0.3)	1.8 (0.2)	2.1 (0.4)	0.79
If I could live my life over, I would change almost nothing	2.3 (0.4)	2.3 (0.5)	2.5 (0.5)	0.72
I feel positive about my future	2.2 (0.4)	2.3 (0.4)	2.8 (0.5)	0.57
I generally feel happy	2.3 (0.3)	2.4 (0.4)	2.9 (0.5)	0.66
I am satisfied with my health	2.5 (0.3)	2.6 (0.3)	3.2 (0.5)	0.31
**Future well‐being**				
Better in a year	52.1	52.7	67.1	0.95
Better in two years	44.6	46.5	61.3	0.74
Better in three years	41.5	45.5	59.1	0.47
**Relative well‐being**				
Same or better off than neighbors	42.7	48.6	63.2	0.24
Same or better off than friends	47.8	51.0	61.0	0.40
**Placement on ladder** c				
Self (1–6)	1.2 (0.1)	1.2 (0.1)	1.6 (0.2)	0.87
Neighbors (1–6)	1.9 (0.3)	1.9 (0.2)	2.0 (0.3)	0.41
Friends (1–6)	1.9 (0.2)	1.9 (0.2)	1.9 (0.3)	0.48
*Observations*	*1,605*	*1,760*	*821*	
*Clusters*	*14*	*15*	*29*	

*Notes*: No significant differences between T and C (*P*‐value < 0.1). *P*‐values are based on tests for the equality of village cluster (VC) means across groups, adjusted for TA stratification.

^a^Range of 8 to 40 from the sum of scale item questions (scored 1 to 5).

^b^Items range from 1 (Strongly disagree) to 5 (Strongly agree).

^c^Ladder ranges from 1 (poor) to 6 (rich).

**Table A6:** Effect estimates of Malawi's SCTP on additional outcome variables and individual Quality of Life (QoL) Scale items

	Additional outcomes^a^			Individual items in QoL Scale^b^							
	Life will be better in a year	Life will be better in three years	Relative wealth: same or better off than friends	In most ways my life is close to ideal	The conditions in my life are excellent	I am satisfied with my life	So far I have gotten the important things I want in life	If I could live my life over I would change almost nothing	I feel positive about my future	I generally feel happy	I am satisfied with my health
				(1)	(2)	(3)	(4)	(5)	(6)	(7)	(8)
Treatment^*^ time	0.15*	0.22***	0.03	0.45***	0.50***	0.52***	0.38**	0.07	0.59***	0.50***	0.36***
	(0.07)	(0.07)	(0.07)	(0.11)	(0.15)	(0.15)	(0.14)	(0.21)	(0.12)	(0.13)	(0.12)
Wild bootstrap *P*‐value	0.068	0.001	0.626	0.000	0.001	0.002	0.024	0.739	0.000	0.000	0.003
Treatment	0.19	0.18	0.33	−0.57	0.35	0.08	0.16	−0.04	0.72	−0.23	−0.44
	(0.43)	(0.41)	(0.32)	(0.90)	(0.97)	(0.83)	(0.79)	(0.80)	(0.93)	(0.98)	(0.80)
Time	0.06	0.01	0.02	0.22***	0.16	0.32***	0.00	0.20	0.19**	0.21**	0.36***
	(0.03)	(0.03)	(0.05)	(0.07)	(0.11)	(0.11)	(0.06)	(0.15)	(0.07)	(0.09)	(0.08)
*Observations*	5,641	5,206	5,826	5,835	5,837	5,837	5,837	5,835	5,829	5,837	5,837
*R^2^*	0.055	0.106	0.014	0.071	0.071	0.081	0.048	0.029	0.101	0.086	0.118

*Notes*: ^*^ *P* < 0.1; ^**^ *P* < 0.05; ^***^ *P* < 0.01. All estimates are for the individual panel and modeled using differences‐in‐differences.

^a^Dependent variables are binary.

^b^Each dependent variable is scored between 1 (strongly disagree) and 5 (strongly agree). Robust standard errors in parentheses clustered at the VC level; Wild *t*‐bootstrap *P*‐value (H_0_ = 0,) are for coefficient on the program impact. Models are fully interacted (controls × treatment indicator) and controls include Demographics (female, age, age squared, ever attended school, chronic illness, married) and Household characteristics (baseline values of household size, total age group categories—0 to 5, 6 to 11, 12 to 17, 65‐plus—and dummies for TA residence).

**Table A7:** Ancillary estimates of the average treatment effects of Malawi's SCTP on measures of subjective well‐being

	Quality of Life Scale			Life will be better in two years			Relative wealth: same or better off than neighbors		
	(1)	(2)	(3)	(4)	(5)	(6)	(7)	(8)	(9)
Treatment	2.95***	3.02***	3.41***	0.18***	0.19***	0.19***	0.06	0.07*	0.12*
	(0.48)	(0.48)	(0.83)	(0.04)	(0.04)	(0.06)	(0.04)	(0.04)	(0.07)
*Wild bootstrap P‐value*	0.000	0.000	0.000	0.000	0.000	0.005	0.140	0.120	0.099
Demographics		X			X			X	
Household characteristics		X			X			X	
Individual fixed effects			X			X			X
Both waves of data			X			X			X
*Observations*	2,919	2,919	5,838	2,455	2,455	5,374	2,907	2,907	5,826
*R^2^*	0.07	0.10	0.14	0.05	0.11	0.06	0.01	0.03	0.01

*Notes*: ^*^ *P* < 0.1; ^***^ *P* < 0.01. All estimates are for the individual panel. Coefficients are for the treatment dummy for the first two columns under each outcome and for the DD coefficient for each last column. Robust standard errors in parentheses clustered at the VC level; Wild *t*‐bootstrap *P*‐value (H_0_ = 0,) for program effect shown below treatment coefficient. Controls include Demographics (female, age, age squared, ever attended school, chronic illness, married) and Household characteristics (baseline values of log per capita expenditure, household size, total age group categories—0 to 5, 6 to 11, 12 to 17, 65‐plus). Dummies for TA residence are included in all models as this was the level of stratification in randomization. Individual fixed effects models drop Demographic controls and Household characteristics drop out because they are defined at baseline.

(A1) $$Y_{it}=\;\alpha_{i}+\beta_{1}T_{i}+\beta_{2}X_{i}+e_{i}$$

(A2) $$Y_{it}=\;\alpha_{i}+\beta_{1}T_{i}*P_{t}+\beta_{2}P_{it}+\beta_{3}X_{it}+e_{it}+v_{i}$$

## References

[pam22044-bib-0001] Attah, R. , Barca, V. , Kardan, A. , MacAuslan, I. , Merttens, F. , & Pellerano, L. (2016). Can social protection affect psychosocial well‐being and why does this matter? Lessons from cash transfers in Sub‐Saharan Africa. Journal of Development Studies, 52, 1115–1131.

[pam22044-bib-0002] Baird, S. , De Hoop, J. , & Özler, B. (2013). Income shocks and adolescent mental health. Journal of Human Resources, 48, 370–403.

[pam22044-bib-0003] Banerjee, A. , & Duflo, E. (2012). Poor economics: A radical rethinking of the way to fight global poverty. New York, NY: PublicAffairs.

[pam22044-bib-0004] Banerjee, A. , Duflo, E. , Goldberg, N. , Karlan, D. , Osei, R. , Parienté, W. , … Udry, C. (2015). A multifaceted program causes lasting progress for the very poor: Evidence from six countries. Science, 348, 1260799. 25977558 10.1126/science.1260799

[pam22044-bib-0005] Banerjee, A. , & Mullainathan, S. (2010). The shape of temptation: Implications for the economic lives of the poor. Cambridge, MA: National Bureau of Economic Research.

[pam22044-bib-0006] Bastagli, F. , Hagen‐Zanker, J. , Harman, L. , Barca, V. , Sturge, G. , Schmidt, T. , & Pellerano, L. (2016). Cash transfers: What does the evidence say? A rigorous review of programme impact and the role of design and implementation features. London, England: Overseas Development Institute.

[pam22044-bib-0007] Beegle, K. , Himelein, K. , & Ravallion, M. (2012). Frame‐of‐reference bias in subjective welfare. Journal of Economic Behavior & Organization, 81, 556–570.

[pam22044-bib-0008] Blattman, C. , Green, E. P. , Jamison, J. , Lehmann, M. C. , & Annan, J. (2016). The returns to microenterprise support among the ultrapoor: A field experiment in postwar Uganda. American Economic Journal: Applied Economics, 8, 35–64.

[pam22044-bib-0009] Cameron, A. C. , Gelbach, J. B. , & Miller, D. L. (2008). Bootstrap‐based improvements for inference with clustered errors. Review of Economics and Statistics, 90, 414–427.

[pam22044-bib-0010] Clark, A. E. , Frijters, P. , & Shields, M. A. (2008). Relative income, happiness, and utility: An explanation for the Easterlin paradox and other puzzles. Journal of Economic Literature, 46, 95–144.

[pam22044-bib-0011] Deaton, A. (2008). Income, health, and well‐being around the world: Evidence from the Gallup World Poll. Journal of Economic Perspectives, 22, 53–72. 19436768 10.1257/jep.22.2.53 PMC2680297

[pam22044-bib-0012] Decancq, K. , & Neumann, D. (2014). Does the choice of well‐being measure matter empirically? An illustration with German data. IZA Discussion Paper No. 8589. Retrieved March 8, 2017, from https://ssrn.com/abstract=2520744.

[pam22044-bib-0013] Diener, E. D. , Emmons, R. A. , Larsen, R. J. , & Griffin, S. (1985). The satisfaction with life scale. Journal of Personality Assessment, 49, 71–75. 16367493 10.1207/s15327752jpa4901_13

[pam22044-bib-0014] Diener, E. , Larsen, R. J. , & Emmons, R. A. (1984). Person × situation interactions: Choice of situations and congruence response models. Journal of Personality and Social Psychology, 47, 580–592. 6491870 10.1037//0022-3514.47.3.580

[pam22044-bib-0015] Diener, E. , Lucas, R. E. , & Oishi, S. (2009). Subjective well‐being: The science of happiness and life satisfaction. Oxford Handbook of Positive Psychology, 2, 187–194.

[pam22044-bib-0016] Dolan, P. , Layard, R. , & Metcalfe, R . (2011). Measuring subjective well‐being for public policy: Recommendations on measures. London, England: Centre for Economic Performance, London School of Economics and Political Science Retrieved January 25, 2017, from http://eprints.lse.ac.uk/47518/.

[pam22044-bib-0017] Dolan, P. , Peasgood, T. , & White, M. (2008). Do we really know what makes us happy? A review of the economic literature on the factors associated with subjective well‐being. Journal of Economic Psychology, 29, 94–122.

[pam22044-bib-0018] Douthitt, R. A. , MacDonald, M. , & Mullis, R. (1992). The relationship between measures of subjective and economic well‐being: A new look. Social Indicators Research, 26, 407–422.

[pam22044-bib-0019] Easterlin, R. A. (1974). Does economic growth improve the human lot? Some empirical evidence. Nations and Households in Economic Growth, 89, 89–125.

[pam22044-bib-0020] Easterlin, R. A. , McVey, L. A. , Switek, M. , Sawangfa, O. , & Zweig, J. S. (2010). The happiness–income paradox revisited. Proceedings of the National Academy of Sciences, 107, 22463–22468. 10.1073/pnas.1015962107 PMC3012515 21149705

[pam22044-bib-0021] Ellis, F. (2012). “We Are All Poor Here”: Economic difference, social divisiveness and targeting cash transfers in Sub‐Saharan Africa. Journal of Development Studies, 48, 201–214.

[pam22044-bib-0022] EU Commission . (2009). GDP and beyond: Measuring progress in a changing world. Brussels, Belgium: Author.

[pam22044-bib-0023] Fafchamps, M. , & Shilpi, F. (2009). Isolation and subjective welfare: Evidence from South Asia. Economic Development and Cultural Change, 57, 641–683.

[pam22044-bib-0024] Folkman, S. (1997). Positive psychological states and coping with severe stress. Social Science & Medicine, 45, 1207–1221. 9381234 10.1016/s0277-9536(97)00040-3

[pam22044-bib-0025] Frey, B. S. , & Stutzer, A. (2002). What can economists learn from happiness research? Journal of Economic Literature, 40, 402–435.

[pam22044-bib-0026] Frijters, P. , Haisken‐DeNew, J. P. , & Shields, M. A. (2004). Money does matter! Evidence from increasing real income and life satisfaction in East Germany following reunification. American Economic Review, 94, 730–740.

[pam22044-bib-0027] Gardner, J. , & Oswald, A. J. (2007). Money and mental well‐being: A longitudinal study of medium‐sized lottery wins. Journal of Health Economics, 26, 49–60. 16949692 10.1016/j.jhealeco.2006.08.004

[pam22044-bib-0028] Gennetian, L. A. , & Shafir, E. (2015). The persistence of poverty in the context of financial instability: A behavioral perspective. Journal of Policy Analysis and Management, 34, 904–936.

[pam22044-bib-0029] Graham, C. , & Behrman, J. R. (2010). How Latin Americans assess their quality of life: Insights and puzzles from novel metrics of wellbeing In Paradox and perception: Measuring quality of life in Latin America (pp. 1–21). Washington, DC: Brookings Institution Press.

[pam22044-bib-0030] Green, E. P. , Blattman, C. , Jamison, J. , & Annan, J. (2016). Does poverty alleviation decrease depression symptoms in post‐conflict settings? A cluster‐randomized trial of microenterprise assistance in Northern Uganda. Global Mental Health, 3, e7. 28596876 10.1017/gmh.2015.28 PMC5314750

[pam22044-bib-0031] Handa, S. , Kilburn, K. , Barrington, C. , Angeles, G. , Mvula, P. , & Tsoka, M. (2013). The Malawi SCT impact evaluation inception report. Chapel Hill, NC: Carolina Population Center at the University of North Carolina.

[pam22044-bib-0032] Handa, S. , Seidenfeld, D. , Davis, B. , Tembo, G. , & the Zambia Cash Transfer Evaluation Team . (2016). The social and productive impacts of Zambia's Child Grant. Journal of Policy Analysis and Management, 35, 357–387. 28690353 10.1002/pam.21892

[pam22044-bib-0033] Haushofer, J. , Reisinger, J. , & Shapiro, J. (2015). Your gain is my pain: Negative psychological externalities of cash transfers. Working paper. Retrived February 2016 from http://files.givewell.org/files/DWDA%202009/Interventions/Cash%20Transfers/Haushofer_Reisinger_and_Shapiro_2015.pdf

[pam22044-bib-0034] Haushofer, J. , & Shapiro, J. (2016). The short‐term impact of unconditional cash transfers to the poor: Experimental evidence from Kenya. Quarterly Journal of Economics, 4, 1973–2042. 10.1093/qje/qjw025 PMC7575201 33087990

[pam22044-bib-0035] Hjelm, L. (2016). The impact of cash transfers on food security. Transfer Project Research Brief 2016–01. Chapel Hill, NC: Carolina Population Center, UNC‐Chapel Hill.

[pam22044-bib-0036] Honorati, M. , Gentilini, U. , & Yemtsov, R. G. (2015). The state of social safety nets 2015. Washington, DC: World Bank Group.

[pam22044-bib-0037] Kahneman, D. (2003). Maps of bounded rationality: Psychology for behavioral economics. American Economic Review, 93, 1449–1475.

[pam22044-bib-0038] Kahneman, D. , & Deaton, A. (2010). High income improves evaluation of life but not emotional well‐being. Proceedings of the National Academy of Sciences, 107, 16489–16493. 10.1073/pnas.1011492107 PMC2944762 20823223

[pam22044-bib-0039] Kahneman, D. , & Krueger, A. B. (2006). Developments in the measurement of subjective well‐being. Journal of Economic Perspectives, 20, 3–24.

[pam22044-bib-0040] Keele, L. , Tingley, D. , & Yamamoto, T. (2015). Identifying mechanisms behind policy interventions via causal mediation analysis. Journal of Policy Analysis and Management, 34, 937–963.

[pam22044-bib-0041] Kobau, R. , Sniezek, J. , Zack, M. , Lucas, R. , & Burns, A. (2010). Wellbeing assessment: An evaluation of well‐being scales for public health and population estimates of well‐being among US adults. Applied Psychology: Health and Wellbeing, 2, 272–297.

[pam22044-bib-0042] Laderchi, C. R. , Saith, R. , & Stewart, F. (2003). Does it matter that we do not agree on the definition of poverty? A comparison of four approaches. Oxford Development Studies, 31, 243–274.

[pam22044-bib-0043] Lloyd‐Sherlock, P. , Barrientos, A. , Moller, V. , & Saboia, J. (2012). Pensions, poverty and wellbeing in later life: Comparative research from South Africa and Brazil. Journal of Aging Studies, 26, 243–252.

[pam22044-bib-0044] Lucas, R. E. , Diener, E. , & Suh, E. (1996). Discriminant validity of well‐being measures. Journal of Personality and Social Psychology, 71, 616–628. 8831165 10.1037//0022-3514.71.3.616

[pam22044-bib-0045] Malawi SCTP Evaluation Team . (2015). Malawi social cash transfer programme midline evaluation report. Chapel Hill, NC: Carolina Population Center at the University of North Carolina.

[pam22044-bib-0046] Miller, C. , Tsoka, M. , & Reichart, K. (2008). Impact evaluation report external evaluation of the Mchinji Social Cash Transfer pilot. Boston, MA: Center for International Health and Development, Boston University School of Public Health.

[pam22044-bib-0047] Ravallion, M. , & Lokshin, M. (2010). Who cares about relative deprivation? Journal of Economic Behavior & Organization, 73, 171–185.

[pam22044-bib-0048] Rayo, L. , & Becker, G. S. (2007). Evolutionary efficiency and happiness. Journal of Political Economy, 115, 302–337.

[pam22044-bib-0049] Rojas, M. (2007). Heterogeneity in the relationship between income and happiness: A conceptual‐referent‐theory explanation. Journal of Economic Psychology, 28, 1–14.

[pam22044-bib-0050] Rojas, M. (2008). Experienced poverty and income poverty in Mexico: A subjective well‐being approach. World Development, 36, 1078–1093.

[pam22044-bib-0051] Rojas, M. (2015). Poverty and people's wellbeing In Glatzer W., Camfield L., Møller V., & Rojas M. (Eds.), Global handbook of quality of life (pp. 317–350). Dordrecht, the Netherlands: Springer.

[pam22044-bib-0052] Samuels, F. , & Stavropoulou, M. (2016). “Being able to breathe again”: The effects of cash transfer programmes on psychosocial wellbeing. Journal of Development Studies, 52, 1099–1114.

[pam22044-bib-0053] Scheier, M. F. , & Carver, C. S. (1985). Optimism, coping, and health: Assessment and implications of generalized outcome expectancies. Health Psychology, 4, 219–247. 4029106 10.1037//0278-6133.4.3.219

[pam22044-bib-0054] Stevenson, B. , & Wolfers, J. (2008). Economic growth and subjective well‐being: Reassessing the Easterlin paradox. Cambridge, MA: National Bureau of Economic Research.

[pam22044-bib-0055] Stiglitz, J. , Sen, A. , & Fitoussi, J. P. (2009). The measurement of economic performance and social progress revisited. Reflections and overview. Commission on the measurement of economic performance and social progress, Paris, France.

[pam22044-bib-0056] The Kenya CT‐OVC Evaluation Team . (2012a). The impact of Kenya's cash transfer for orphans and vulnerable children on human capital. Journal of Development Effectiveness, 4, 38–49.

[pam22044-bib-0057] The Kenya CT‐OVC Evaluation Team . (2012b). The impact of the Kenya cash transfer program for orphans and vulnerable children on household spending. Journal of Development Effectiveness, 4, 9–37.

[pam22044-bib-0058] Watson, D. , Clark, L. A. , & Carey, G. (1988). Positive and negative affectivity and their relation to anxiety and depressive disorders. Journal of Abnormal Psychology, 97, 346–353. 3192830 10.1037//0021-843x.97.3.346

[pam22044-bib-0059] Weimann, J. , Knabe, A. , & Schöb, R. (2015). Measuring happiness: The economics of well‐being. Cambridge, MA: The MIT Press.

[pam22044-bib-0060] World Health Organization (WHO) . (1998). The World Health Organization quality of life assessment (WHOQOL): Development and general psychometric properties. Social Science & Medicine, 46, 1569–1585. 9672396 10.1016/s0277-9536(98)00009-4

